# Indoor and Outdoor Social Alarms: Understanding Users' Perspectives

**DOI:** 10.2196/mhealth.2730

**Published:** 2014-03-07

**Authors:** Marie Sjölinder, Anneli Avatare Nöu

**Affiliations:** ^1^SICS Swedish ICTKistaSweden

**Keywords:** health services for the aged, technology, man-machine systems, computer communication networks, caregivers, social alarm, security, safety

## Abstract

The elderly population is increasing and there is a need to provide care and safety at a high level with limited resources. New social alarm solutions may contribute to safety and independence for many elderly. However, it is important to understand the needs within the user group. This work studied social alarms in a broad sense and from several user perspectives. In the first study, social alarm use and its aspects were investigated. To understand where there may be problems and weaknesses, users, caregivers, managers of municipalities, and personnel at alarm centers were interviewed. The interviews helped identify a number of problems. For municipalities, the processes of procuring new alarms and managing their organization were found to be complex. The effect of this was that the same social alarm systems had been ordered over and over again without taking into account new user needs or new technical solutions. For alarm users, one large problem was that the alarms had very limited reach and were designed for indoor use only. This has resulted in users hesitating to leave their homes, which in turn has negative effects due to lack of physical activity and fewer social contacts. One important result from the first study was the need for a social alarm solution that worked outdoors. In a second study, needs regarding outdoor social alarms were investigated. The results from this study showed that wearable outdoor alarms must be easy to use, provide communication, and be well designed. Finally, these alarms must work both indoors and outdoors, and the user should not have to worry about where he/she is or who is acting on an alarm.

## Introduction

### Safety

The elderly population is increasing and there will be a growing number of elderly living in their own homes. To be able to live an independent and active life with social interaction, elderly people need to feel safe both indoors and outdoors. One way to increase the feeling of safety for them is through social alarms. A variety of alarms exists, both for indoor and outdoor use.

The feeling of being safe is very complex and can be viewed from many perspectives. Social alarm systems are very fragile because the entire chain, from when an alarm holder presses the alarm button until home care staff arrives, must constantly operate. If it fails once, then the feeling of being safe is lost [1]. To discover critical aspects, it is important to get an overall view of the social alarm area, especially the alarm system chain. It is important to understand how the alarm holder experiences security and how it might be affected by different circumstances.

### Indoor Alarm

**Figure 1:**
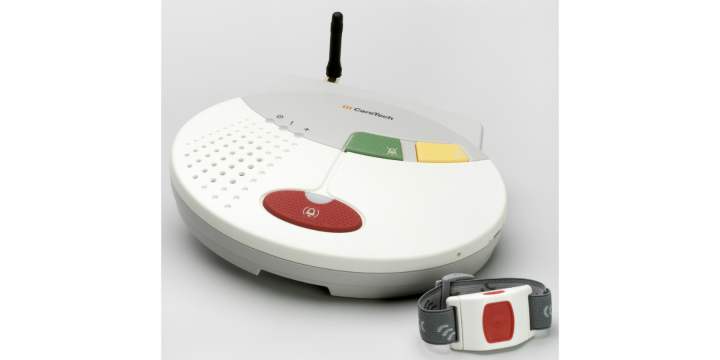
A social alarm – base unit and an alarm button.

A social alarm (Figure 1) is an alarm device that is installed in a user's home and makes it possible for a user to call for help in urgent situations, such as if the person has fallen. The alarm often consists of a base unit and care phone, which is connected to the analogue telephone network or via the digital infrastructure in an apartment or house. An alarm button, which is worn on a necklace or around the wrist, is connected to the base unit. When a user presses the alarm button, a signal (alarm) is sent to an alarm receiver, home care staff, or a relative. Social alarms also have a speech function at the base unit that makes it possible for the person who raised the alarm to talk to the alarm receiver.

In Sweden, social alarms are provided both to people living in ordinary housing and in nursing homes. The municipalities usually provide social alarms. Traditional social alarms are connected to an alarm center that forwards the alarm to the home care or in some cases to a relative or neighbor.

The use of social alarms is similar in many countries. In the United States, for example, social alarms are called personal emergency response system (PERS) and are available in almost all parts of the country. There are both national and local providers, including private companies, hospitals, and social service agencies. PERS acts similarly to social alarms in Sweden, but usually the alarm center contacts a neighbor or a relative; it is the alarm holder who chooses which person to call.

Regardless of differences in organization around the alarms, the most important aspect is the feeling of being safe. In several studies from the United States, Porter et al [2,3] showed that the users wanted PERS to feel safe, knowing they would receive help if anything should happen. In England, research regarding alarms and safety has provided the same result, that is, increased quality of life and increased opportunities for safe living [4]. In Sweden, Lindén [5] conducted a study in which the aim was to investigate seniors' experiences and perceptions of social alarms. This study reported similar results as previous studies and also showed that social alarms have become a part of users’ daily lives and not perceived as an intrusion or something negative.

For the elderly, it is important to feel safe in their homes. According to the results from the described studies, the elderly usually felt safe in the home when they had a social alarm. However, there is also a need for the elderly to be more active and feel safe outdoors as well. Because of this, we wanted to go one step further and investigate needs and implications for the use of outdoor alarms.

### Outdoor Alarm

Traditional social alarms only work in a home environment. For this reason, older people hesitate to leave their homes because they do not feel safe. This was shown in a study by Boström et al [6], which showed that social alarms could create a feeling of insecurity rather than security, and that it could restrict the elderly from moving around free in society. The elderly felt that they did not dare go out because the social alarm only had a reach within the house or just around the house. This led to limited integration into society and less social interaction. Physical activity and social contacts are important for health and disease prevention. An outdoor alarm could help people to feel safe even when outdoors, and thereby enable them to have an active life with better health and improved quality of life. Taylor et al [7,8] have done several studies within the social alarm area or as they call it “community alarm service.” In one of the surveys, changes to the social alarm were suggested. The most frequent comments were that the social alarm should send a call for help if the wearer falls and that it should work outside the home. Taylor et al [7] presented a design list for the next generation of the social alarm and the alarm button. One of the design features was “long-range operation” which means that the social alarm would work outside home and equipped with a global positioning system (GPS) and mobile communications.

There are several outdoor alarms on the market that can locate a person via GPS. This kind of social alarm enables an alarm receiver to see where the person is located. With this service, you can enter a geographic security zone for a person. When the person goes outside the zone, an alarm is sent to the alarm receiver's mobile phone to indicate the user's position. Relatives can either call or seek the user at the given position if they become anxious. This type of alarm can be used passively (violation of the security zone as described above) and actively by the use of an alarm button (which a user can press to call for assistance). These solutions can also have other functionalities such as detecting falls or lack of action (a passive alarm that sends alarm calls based on user actions or absence of those). For example, within the project MyHealth@Age [9], a mobile phone-based solution that consisted of both active and passive alarm functionality was developed.

One problem with outdoor alarms is that they are based on different technologies than the indoor alarms and are not integrated into the same alarm solution. A social alarm that can handle both indoor and outdoor environments in the same alarm solution is something that both the elderly and their relatives have requested [1]. However, these solutions are difficult to find on the market. This means that a person having a traditional social alarm at home needs to bring another alarm with him or her when he or she goes out. When it comes to alarms that can be positioned, users can be anywhere when the alarm is set off. This leads to challenges with respect to organization regarding acting on alarms, because home care staff usually only come and assist when the alarm holder has alerted in his or her home with a traditional social alarm.

### Needs and Challenges in the Social Alarm Area

The social alarm area has a strong tradition regarding functionality and design. In recent years, however, the area started to undergo changes that involved both challenges and opportunities. One challenge is, for example, the replacement of the analogue network with a digital infrastructure. Sweden has reached far within the digital social alarm area in terms of the development of alarms, the use of the digital infrastructure for the alarms, and management of the alarm solutions. We will not describe this infrastructure and the implications for the alarms in this paper but it opens up new possibilities in the field of social alarms that can be helpful to meet user needs in the future.

Two studies are presented in this paper. To understand how the future alarm area should work, it is important to address the social alarm area in a broad sense and from several perspectives. In the first study, we investigated needs and attitudes among all relevant stakeholders. The study also examined the interaction between the stakeholders and how the entire alarm chain worked. From the alarm holder's perspective, needs and attitudes toward safety were investigated. The method we used addressed user needs in an explorative way and from a broad perspective.

Based on the results from the first study, which showed a need for increased mobility, the next step was to investigate user needs with respect to alarms that work outdoors. There are challenges that need to be addressed to meet the needs and to find solutions to the organization around outdoor social alarms.

Social alarms work similarly in the rest of Europe and in the United States [10]. Results and conclusions presented here could also be relevant in other countries.

The aim with this work was to understand how social alarms were used, and identify shortcomings and how the users experienced safety. Further, this paper addresses future functionality and better design. We provide suggestions for requirements to be met in future indoor and outdoor social alarm solutions.

## Study 1: Indoor Alarms and User Needs

### Objective

**Figure 2:**
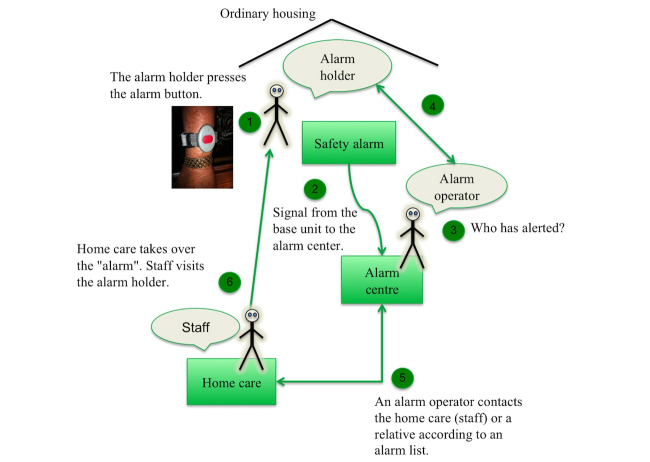
The Alarm Chain - it starts when an alarm holder presses the alarm button and ends when staff from home care visits the alarm holder.

In this study, the objective was to investigate the use of the alarms and the alarm chain (Figure 2) from a user perspective. It was important to obtain a view of the social alarm processes—from procurement to when an alarm holder presses the alarm button until home care staff visits the alarm holder and assists—to understand what might affect/increase security.

### Method

#### Overview

This study was divided into two phases, which were conducted sequentially. In the first phase, we started with an overview of the systems and investigated existing alarm solutions from a user perspective. Finally, previous purchases made by municipalities were studied.

In the second phase, four municipalities participated. It was important to understand attitudes toward the alarms and how they were used. Further, we wanted to understand the organization around the social alarms, and how this differed between urban and rural areas. Open-ended interviews with senior users and with staff from the municipalities were conducted. The process of procurement and the requirements around the social alarm were investigated by interviews with responsible personnel. During the interviews, the researchers took notes. A qualitative analysis was conducted based on a grounded theory approach [11].

#### Phase 1

To begin, a comparison was made of 15 procurements (from municipalities in Sweden 2011) for social alarms and alarm receiving for ordinary housing.

The aim was to get a better understanding of the content in these procurements so that we could develop relevant questions for the municipalities. In particular, we investigated to which extent the focus was placed on users’ needs in the procurements.

In parallel, a survey was conducted to develop an overview of the existing alarm solutions from a user perspective. Based on these results, we could then take it a step further and highlight weaknesses and what municipalities should consider when procuring social alarms.

The entire chain (Figure 2) must constantly work. If it fails once, then the feeling of security is broken [1]. In our work, we focused on use and user aspects of these processes. There are several actors involved and there are complex processes with respect to procuring new alarms and managing the organization around them. To understand the social alarm area, key members of the alarm chain were interviewed, including alarm users, home care staff/managers, and operators at alarm center.

#### Phase 2

**Figure 3:**
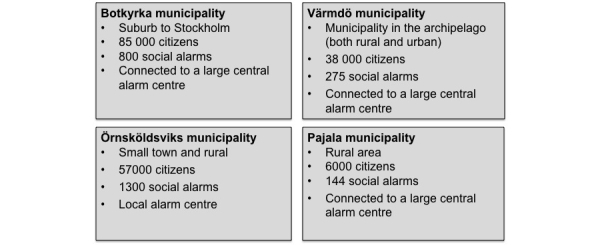
Field studies in four municipalities.

In this phase, the work was conducted as a collaboration between SICS Swedish ICT and four municipalities in Sweden . The municipalities were selected to reflect the social alarm field from diverse needs and different circumstances (Figure 3). Botkyrka is an urban municipality with challenges of different needs based on many different cultures. Värmdö is both urban municipality with its conditions, but also a rural municipality because the municipality covers large parts of the archipelago of Stockholm. Furthermore, Örnsköldsvik represents both rural and smaller city. Pajala has participated as a rural municipality with special circumstances this means. Open-ended interviews were used to understand how municipalities procured social alarms and to get an overall perspective of the alarm chain. One further purpose of the qualitative interviews was to detect lesser known phenomena and properties in the social alarm area. Most of the interviews were conducted at the municipality offices or at the home care facilities. All interviews with staff in Pajala municipality were conducted by telephone because of the distance. Interviews with some of the elderly alarm holders (in all municipalities) were conducted by telephone. Notes were taken during the field studies.

In the study, both needs of users (of social alarm) and requirements from municipalities were investigated. Interviews were used to understand how municipalities procure social alarms today and to get an overall perspective of the alarm chain. The interviews were conducted with managers in the municipalities, personnel at alarm centers, and alarm holders. Furthermore, alarm users in ordinary housing were interviewed. Alarm operators and other personnel were interviewed at alarm centers. Approximately 2-3 alarm holders, in each municipality, 1-2 heads of unit, staff members, and alarm operators were interviewed. We had a contact person at each municipality who arranged with participants for the interviews.

In 2 of the municipalities, some alarm holders were interviewed in their homes. The interviews were conducted by telephone and included open-ended questions that focused on the social alarm (the alarm button and care phone/speakerphone), situations when they alerted, if it was indicated that the alarm had been sent away, and advantages/disadvantages with the social alarm.

In all municipalities, the unit manager for home care (for the elderly) was asked open-ended interview questions about how social alarms were handled, procurement/purchasing, any problems with social alarms, future prospects, etc.

In all municipalities, the staff within home care was interviewed; open-ended questions were about how they experienced the social alarms and how it worked, how their job was, how the alarm was received from the alarm center, and how the staff acted when they received an alarm. It was important to know how the staff thought that the management of the social alarm system worked and how they felt it worked for alarm holders to alert.

In 1 of the municipalities, alarm operators were interviewed at the municipality's own alarm center. This was a relatively small alarm center, but the principle of how alarms were received and forwarded to responsible staff at the home care were the same. Observations of the work were also conducted at the alarm center.

In all municipalities, people who worked with the procurement of social alarms were interviewed with open-ended questions. The aim was also to gain a broader understanding of how they defined the "shall requirements." There were also questions about the dialogue between the municipality and companies within the social alarm area.

Because the aim of the project was to address the social alarm field from a holistic perspective, it was important to interview people with different responsibilities within the social alarm process and users of the alarm. To understand what worked well, what changes needed to be made, where there were problems, lack of knowledge, etc, it was important to gain an understanding of the whole process.

The results presented below are a summary of the most frequent topics from the material.

### Results and Conclusions

Some general observations based on the interviews with the municipalities are highlighted here. It should be noted that all 4 municipalities that participated had similar problems and shortcomings. The municipalities had inadequate knowledge about the new technology in the social alarm field. It was shown that it was a complex process to procure social alarms and extensive knowledge within the municipalities was required. Three of the municipalities trusted the suppliers regarding requirements on the alarms. For the municipalities, it was difficult to know what products that exist on the market, and difficult to know the needs of the users. As a result, the same social alarm systems were ordered repeatedly without taking into account new user needs or new technical solutions. Two of the municipalities said that they needed more knowledge about the new digital social alarms to procure social alarms of good quality. The other 2 municipalities had made more progress in gaining knowledge about the new digital social alarms. One of the municipalities had employed a person with the skills, and the other one participated in a larger project on digital social alarms together with other municipalities in northern Sweden.

Based on the study of the previous procurements and interviews with the municipalities, it was found that many municipalities prioritized to meet the basic specifications at the lowest price. This has the implication that innovative solutions, from small companies, have difficulties to enter the market. These products are unable to compete for the lowest price. Smaller companies are also excluded when the municipalities repeatedly return to the larger companies for products similar to what they already have

In the future, it will be important to analyze the users' need for security and how this need could be met by new solutions. It will also be important for the municipalities to ensure that the safety and quality of social alarms really address the users’ needs, both with respect to alarm holders and personnel.

Several problems were revealed during the interviews with the users. One large problem was that the alarms had a very limited reach and were designed for indoor use only. If a user was too far away from the base unit, the alarm did not work. Furthermore, in the traditional indoor alarms, the speaker is located in the base unit and the user had to be quite close to the unit to communicate with the alarm center. The ability to have voice communication via the alarm unit (which could be worn on the wrist) would remedy this problem. This would also increase the users' mobility indoors. Improvements relating to feedback were mentioned, for example, different feedback about when the alarm was sent and when the alarm was received by the alarm center or by home care staff. The problems regarding the traditional social alarms in terms of being unable to use the alarm outdoors had the effect that some elderly people hesitated to leave their homes. In the analysis of the interviews, we could see there was a great need for an outdoor alarm. No existing alarms can handle both traditional indoor alarm usage and outdoor alarm usage in the same solution. The explanation to this is that the alarm solutions are based on different technologies and different organizations in terms of acting on alarms.

## Study 2: Outdoor Alarms and User Needs

### Objective

For the elderly population to live an active life and exercise in a safe way, social alarms must operate outdoors. The aim of this study was to investigate needs among elderly users and their relatives regarding outdoor social alarms. In this paper, we investigated appearance, functionality, and aspects related to responsibility and payment.

### Method

In this study, the material was gathered in two ways through focus groups and open-ended interviews. Focus groups were used to gather new ideas from a broad perspective. The aim was to encourage the participants to evolve new ideas together with others. Interviews were chosen to detect phenomena, properties, and meanings of using outdoor social alarms with respect to safety. The interviews were conducted individually to give participants the opportunity to express their views without the influence of others.

In the interviews, 15 participants from 3 user categories were included: elderly, middle-aged people who took care of their elderly relatives, and young people who helped a grandfather or a grandmother. The questions in the interviews addressed attitudes toward safety and the use of outdoor social alarms. Furthermore, we asked what the participants thought about how and by whom the alarms should be received and acted upon.

Two focus group sessions were carried out, one with elderly people, and one with middle-aged people taking care of one or more elderly relatives. The aim with the sessions was to gather material regarding needs, functionality, and appearance of outdoor alarms. The work in the focus groups started with broad questions regarding the need for safety and social alarms. In the next step, 3 existing alarms were showed to the participants. Each of these alarms solved different aspects of the need for safety. After each presentation, the alarm was discussed based on its advantages and disadvantages, and a wish list was created. The list consisted of features the participants wanted in this kind of alarm. The participants had ideas on new functionality that they thought the alarms lacked. They also pointed out functionality that they thought could be excluded in alarms. As a final step, the participants were asked to prioritize among the functionalities that were described. The result of the session was a list of functionalities, a wish list, for outdoor social alarms.

During the focus group and the interviews, the researchers took notes. The material from the focus group and the interviews was analyzed and categorized with a grounded theory approach [11]. The results presented below are a summary of the most frequent topics from the material.

### Results and Conclusions

When adding outdoor functionality, it becomes much more complex regarding receiving and acting on alarms. In the interviews, the participants were asked about the relatives’ role in receiving alarms. One important result from the interviews was that the relatives were reluctant to receive the alarm calls. Both middle-aged and young relatives wanted to be there for their elderly relative. However, the responsibility of receiving and acting on alarm calls was too great with too many difficult decisions to make. The participants thought that the alarm primary should be connected to an alarm center, but that the relatives should be notified when an alarm call had been made.

In the focus group sessions, both the elderly participants and the relatives pointed out that the wearable alarm unit must be something other than a mobile phone. It has to be a device that would not be forgotten as easily as a mobile phone. It should also have a separate alarm button that is easy to see and press. Even though the alarm should be something other than a mobile phone, the elderly participants pointed out the importance of being able to speak/communicate with the receiver when they had raised the alarm.

The lack of feedback reduced the feeling of safety and increased the uncertainty regarding whether someone would help. A solution suggested by the participants, was to receive differently labeled information if someone actually had received or acted on an alarm call. However, as pointed out by the relatives, it might be difficult to understand too many kinds of feedback,. The feedback must be easy to understand if it should be assigned to all actions in terms of send, receive, and act on alarms. For example, different buttons and/or different lights could be used. The buttons could also have pictures for those who have difficulty remembering which button to press.

When it comes to the design of the alarms, both the elderly and the relatives thought that it was important to make the alarms more personal. For example, it should be possible to choose among different styles and colors. The relatives also pointed out the importance of personalizing the alarm with respect to functionalities.

Finally, we discussed integrity with the participants. In line with previous research [12], none of the participants thought that the use of their geographical location was an invasion of their privacy. The benefits were seen as far greater than the disadvantages to be located using GPS.

## Conclusions and Future Challenges

To summarize the results from the two studies, it was difficult for the municipality to know/understand user needs. The technology was complex and required considerable expertise in the area. There were usually no methods within the municipalities to include user needs in the process when the social alarms were procured. Mangers for social alarms within the municipalities need more knowledge about the technology and user needs to be able to offer tomorrow's new technology to its population. Better dialogue with users about their needs, and new methods in gathering these needs are required to be successful in introducing new types of alarms.

The results showed that the experience of safety did not correspond with the needs that the elderly had. Similar results were also found in other studies [1-5]. One limitation with today's traditional social alarms is that the user only can alert from his or her home and not outdoors. This means that many elderly do not come out on walks because they feel unsafe outside their homes. With aging usually comes memory loss, and it can have devastating effects on the quality of life for older people. Ertel et al [13] found evidence that elderly people in the United States who had an active social life had a slower rate of memory decline.

We believe that to increase the elderly’s quality of life, social alarms also need to operate outdoors. In our studies, we found that there was a great need for outdoor alarms among both users and relatives. Relatives requested alarms with functionality that handled the situations in which the residents risked ending up in danger if he/she left his or her home. In particular, families with an elderly relative with dementia who lived in his or her home felt great insecurity regarding this.

Today, there are examples of outdoor alarms that communicate via mobile networks and have GPS receivers. These alarms also have voice communications, which means that an alarm receiver can talk with the person who has alerted. When adding outdoor functionality, it becomes much more complex regarding receiving and acting on alarms. The municipalities don’t always have resources and routines to take care of this type of alarm. One important result from the interviews showed that it was not obvious that the relatives would like to receive the alarm calls. Both middle-aged and young relatives wanted to be there for their elderly relative, but to be responsible for receiving and acting on alarm calls was too great of a responsibility with too many difficult decisions to make. Participants indicated that the wearable alarm unit must be something other than a mobile phone; that is, it should be a device that will not be forgotten as easily as a mobile phone.

To conclude, there was a great need for alarms that could handle both indoor and outdoor environments. Both our results and other studies indicate this [6-8]. The elderly and their families requested social alarms that could handle both indoor and outdoor environments in the same alarm system. One problem with current indoor and outdoor alarms is that they are based on different technologies and are not integrated into the same alarm solution. With an increasing elderly population and fewer resources, new types of technology solutions are needed for older people to feel safe even when outdoors, which is a prerequisite for an active life with better health and improved quality of life.

Future work will include development of a new combined alarm solution. One important feature will be that the alarm solution consists of a base unit in the home and an alarm device that a user can wear both indoors and outdoors.

Furthermore, the alarm solution should consist of functionalities such as an automatic switch between indoor and outdoor position for increasing battery capacity. When a user is at home, the alarm unit communicates with the base unit just like a traditional alarm and when the alarm holder leaves home, the alarm unit switches to a global system for mobile communications mode with GPS functionality. The wearable unit should have a speaker for communication with the alarm receiver and an alarm button. For outdoor use, it should be possible to use services such as “geofencing” [14] for setting up a geographical zone based on GPS coordinates, and if a user (the wearable unit) crosses the border of this area, an alarm is activated.

Development of such an alarm solution entails a number of challenges, particularly regarding hardware and battery capacity. Furthermore, for these solutions to work, new ways of relating to both the alarm receiving and responsibilities, and for payment, are required. We believe that new ways of allocating these aspects between the private and the public will be required. Despite of these challenges, it is essential that we approach the task. To succeed in designing this type of alarm, cooperation among companies that develop social alarms, municipalities, home care, and end users is needed. However, the most important issue is, and will be, to address the different user groups’ needs and to involve the users in every user-related aspect of development.

## Abbreviations

GPS: global positioning system
PERS: personal emergency response system
